# *Siparuna gesnerioides* and *Siparuna guianensis* Essential Oils in *Aedes aegypti* Control: Phytoanalysis, Molecular Insights for Larvicidal Activity and Selectivity to Non-Target Organisms

**DOI:** 10.3390/plants14091322

**Published:** 2025-04-27

**Authors:** Milton L. Montaño-Campaz, Eugenio E. Oliveira, Beatriz Toro-Restrepo, Tito Bacca, Carolina Feuillet-Hurtado, Javier G. Mantilla Afanador, Renata Pereira Lopes Moreira, Luiza Alves Mendes, Raimundo Wagner S. Aguiar, Lucimar G. Dias

**Affiliations:** 1 Centro de Bioinformática y Biología Computacional de Colombia—BIOS, Ecoparque Los Yarumos, Manizales 170002, Caldas, Colombia; milt-m@hotmail.com; 2 Departamento de Entomologia, Universidade Federal de Viçosa, Viçosa 36570-900, MG, Brazil; 3 Grupo de Investigación Bionat, Facultad de Ciencias Exactas y Naturales, Universidad de Caldas, Manizales 170001, Caldas, Colombia; 4 Facultad de Ingeniería Agronómica, Universidad del Tolima, Ibagué 730001, Tolima, Colombia; 5 Research Institute in Microbiology and Agroindustrial Biotechnology, Universidad Católica de Manizales, Carrera 23, 60–63, Manizales 170002, Caldas, Colombia; 6 Departamento de Química, Universidade Federal de Viçosa, Viçosa 36570-900, MG, Brazil; 7 Programa de Pós-Graduação em Biotecnologia, Universidade Federal do Tocantins, Gurupi 77402-970, TO, Brazil

**Keywords:** plant-based biorational insecticides, sesquiterpenes, mosquito predators, *Belostoma anurum*

## Abstract

Synthetic insecticides are widely used against mosquitoes, but misuse has led to environmental and health concerns. Plant-derived alternatives, such as essential oils, seem to offer a safer option, minimizing these problems without compromising efficacy. In this study, we evaluated the essential oil from *Siparuna gesnerioides* (Kunth) A.DC., a Neotropical plant, for its effectiveness in controlling *Aedes* (*Stegomyia*) *aegypti* (Linnaeus) larvae, a major vector of human diseases. We first assessed the phytochemistry of the essential oil and used in silico approaches to predict potential physiological targets of its larvicidal activities. Selectivity assays were conducted with *Belostoma anurum* (Herrich-Schäffer), a non-target predatory water bug. The major constituents of *S. gesnerioides* essential oil were γ-elemene (45.8%) and germacrene D (43.8%). This essential oil effectively killed larvae from both susceptible and resistant mosquito strains (LC_50_ = 0.070 μg/mL). However, such concentrations killed more than 80% of *B. anurum* nymphs. Molecular modeling suggested that the essential oil major components (γ-elemene and germacrene D) interact stably with mosquito acetylcholinesterases (AChEs), indicating a potential mechanism of action. Our results reinforce the potential of *Siparuna* essential oils in mosquito control. Nevertheless, the non-selective impact on mosquito predators, as seen with *S. gesnerioides*, highlights the need for caution in field applications.

## 1. Introduction

*Aedes aegypti* (Diptera: Culicidae) is a relevant vector for diseases such as yellow fever, dengue, Zika, chikungunya, and Mayaro fever, found across the Americas, Africa, and Asia [1,2,3]. Conventional control strategies, including the elimination of breeding sites and the application of insecticides, have been pivotal in managing these vector-borne diseases [4,5]. However, despite their effectiveness, these methods have drawn criticism due to the rise in global prevalence of *A. aegypti* and the development of resistance in mosquito populations [6,7]. Furthermore, the widespread use of insecticides has raised concerns about their detrimental impacts on human health and the environment, especially on non-target fauna [8,9,10,11].

In response to these challenges, efforts have been made to explore alternative approaches to achieve more sustainable mosquito control. Among these approaches, biorational products (e.g., plant extracts and essential oils) derived from botanical species have gained attention for their potential to control insect vectors with reduced risks compared to synthetic chemicals [12,13]. For instance, *A. aegypti* can be effectively managed using plant-based essential oils, although the efficacy of these biorational insecticides can vary depending on genetic and environmental factors that affects their chemical composition [14,15,16,17,18].

The Neotropical region, rich in diverse flora, remains an underutilized source of biologically active substances. Plants of the genus *Siparuna* (Siparunaceae), commonly known as Negramina, are widespread in South America and have been traditionally used in folk medicine [19,20,21,22]. Despite this plant genus contains a diverse range of species [23,24], most of the insecticidal potential investigations has focused on the *Siparuna guianensis* Aubl., whose essential oils (alone or in nanocomposites) are shown to be effective in controlling pests such ticks [16], moths [25,26], aphids [27], and mosquitoes [17,28,29] with low toxicity to non-target organisms [27,28,29].

In this study, we assessed the chemical composition and toxicity of essential oils extracted from *S. gesnerioides* (Kunth) A.DC. and compared these parameters with the essential oil extracted from *S. guianensis* collected in the same region. As undesired effects of plant-based biorational products are not only related to synthetic insecticides [30,31,32], we further analyzed the potential undesired effects of these essential oils on the giant water bugs, *Belostoma anurum* (Hemiptera: Belostomatidae), whose nymphs are generalist predators, representing a natural control agent for mosquitoes [9,33,34,35], and have been shown to be indirectly affected by synthetic and plant-based insecticides [9,35,36]. Furthermore, by applying in silico predictions, we further investigate the molecular interaction between the major constituents of the *Siparuna* essential oils and the *A. aegypti* acetylcholinesterases (AChEs), a detoxifying enzyme whose disrupted activities can lead to neurotransmission malfunctions in mosquitoes and have been target by several essential oils.

## 2. Results

### 2.1. Yield and Chemical Composition of Essential Oils

**Figure 1:**
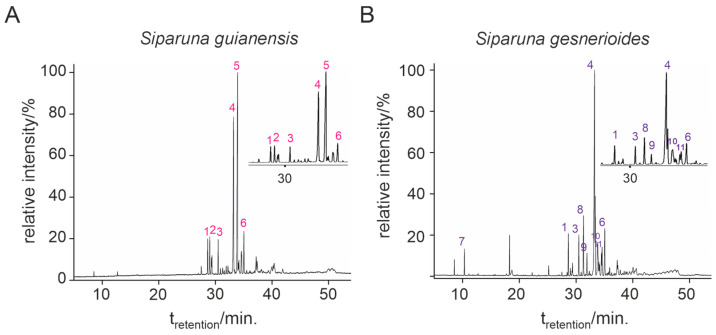
Chemical composition of each of the constituents of *Siparuna guianensis* (**A**) and *Siparuna gesnerioides* (**B**) essential oils by gas chromatography coupled to mass spectrometry.

*Siparuna gesnerioides* presented the highest essential oil yield (0.384%) compared to *S. guianensis* (0.210%). Both essential oils presented a viscous liquid of bright yellow color, *sui generis* odor, and less dense than water. Qualitative and quantitative variations in chemical composition were observed between both essential oils. Six compounds were identified for *S. guianensis* (Table 1, Figure 1A), which represented 100% of the essential oil composition. For *S. gesnerioides*, nine compounds were identified (Table 1, Figure 1B), which represented 94.8% of the essential oil composition. All essential oils presented more than 94% of sesquiterpenes in their composition and very low contents of monoterpenes.

### 2.2. Toxicity of Siparuna Essential Oils Against Aedes aegypti Larvae

**Figure 2:**
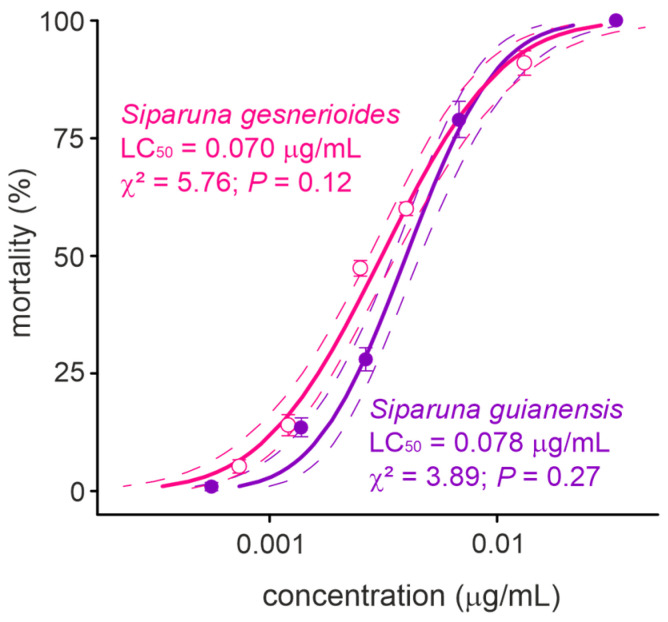
Toxicity (24 h of exposure) of *Siparuna gesnerioides* and *Siparuna guianensis* to *Aedes aegypti* larvae. Solid lines represent the estimated mortality values by the PROBIT model. Dotted lines represent the 95% confidence intervals and symbols shows the mean (±SEM) obtained for four replicates.

**Figure 3:**
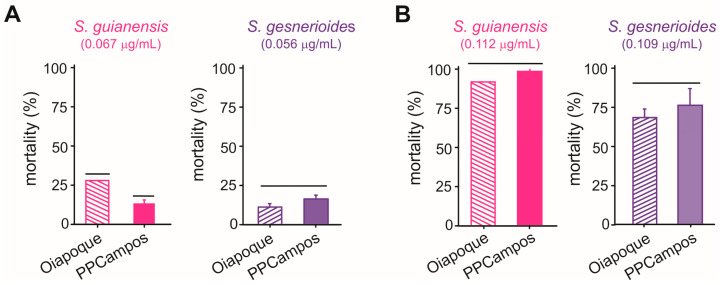
Mortality (%) of *Aedes aegypti* larvae of insecticide-resistant (Oiapoque) and insecticide-susceptible (PPCampos) strains when exposed to *Siparuna guianensis* and *Siparuna gesnerioides* at their LC_25_ (**A**) and LC_80_ (**B**) values. Bars grouped at the same horizontal lines indicate the absence of significant differences according to Tukey’s HSD test (*p* < 0.05). Mortality was recorded over a 24 h period.

The concentration-mortality results obtained for *S. guianensis* (n = 700; χ^2^ = 3.89; *p* = 0.27) and *S. gesnerioides* (n = 700; χ^2^ = 5.76; *p* = 0.12) on *A. aegypti* larvae after a 24 h exposure were successfully fit to a Probit model (Figure 2), which allowed the estimation of the lethal concentrations for each of the *Siparuna* essential oils on the PPCampos strain. *Aedes aegypti* larvae were similarly killed by *S. gesnerioides* (LC_50_ = 0.070 [0.066–0.074] μg/mL) and *S. guianensis* (LC_50_ = 0.078 [0.073–0.082] μg/mL) (Table 2; Figure 2). Both *Siparuna* essential oils were similarly toxic to insecticide-susceptible and -resistant larvae of *A. aegypti* (Figure 3). Interestingly, the exposure to the LC_25_ (0.067 μg/mL) of *S. guianensis* essential oil killed significantly more insecticide-resistant larvae compared to susceptible mosquito larvae (Figure 3). Such differential activities were not presented to larvae exposed to the LC_25_ (0.056 μg/mL) of *S. gesnerioides* essential oil (Figure 3). At higher essential oil concentrations (i.e., LC_80_) there was no significant difference in terms of mortality of insecticide-susceptible and resistant larvae (Figure 3).

### 2.3. Molecular Interactions Between Essential Oil Major Constituents and Aedes aegypti Acetylcholinesterases

**Figure 4:**
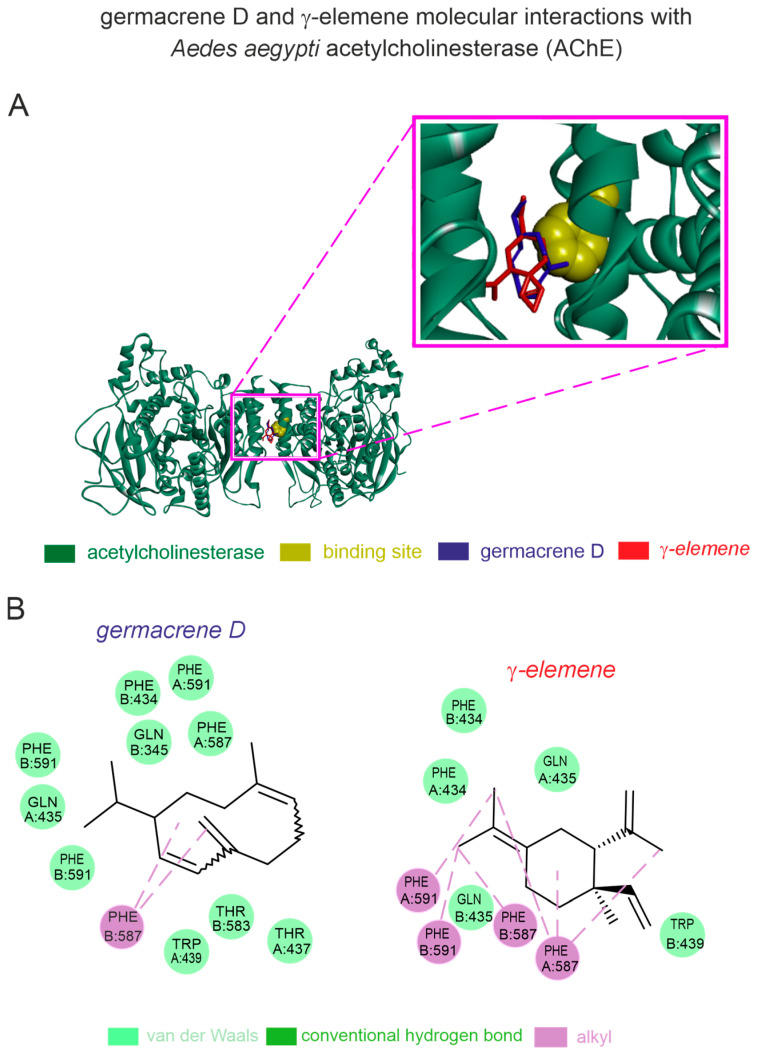
In silico prediction of interactions of germacrene-D and γ-elemene with acetylcholinesterase (AChE) from the dengue-transmitting mosquito. (**A**) Protein 3D structure of the mosquito-related AChE with germacrene-D and γ-elemene molecules; (**B**) 2D interaction maps of AChE interaction sites with germacrene-D and γ-elemene.

Based on our in silico computational predictions, the germacrene D and γ-elemene present in can interact with *A. aegypti* AChE through stable binding pockets. The homology modeling of the *A. aegypti* AChE showed a high-quality structure with 93% Ramachandran favored values and a QMEAN factor of −1.04. Both germacrene D (−6.8 kcal [−28.45 kJ]/mol) and γ-elemene (−6.7 kcal [−28.03 kJ]/mol) exhibited stronger binding affinities to the *A. aegypti* AChE. The germacrene D—*A. aegypti* AChE complex displayed van der Waals interactions with TRP439, THR583, THR437, PHE434, GLN435, PHE587, PHE434, and PHE591, as well as alkyl interactions with PHE587 (Figure 4). The γ-elemene complex showed van der Waals interactions with TRP439, GLN435, and PHE434, and pi-alkyl interactions with PHE587 and PHE591 (Figure 4).

### 2.4. Selectivity of Siparuna Essential Oils to Belostoma anurum Nymphs

**Figure 5:**
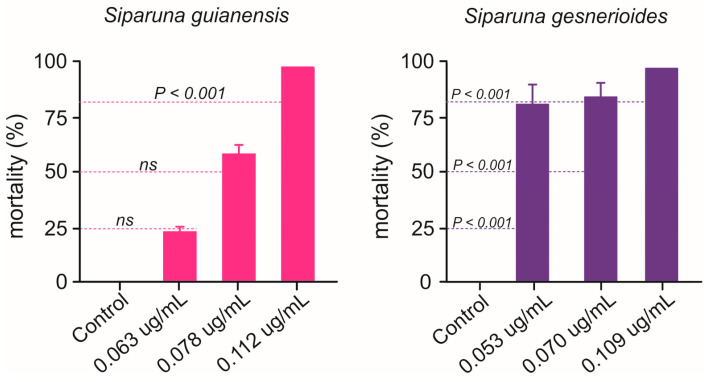
Mortality (%) of Belostoma anurum exposed to concentrations (LC_25_, LC_50_, and LC_80_) of essential oils of *Siparuna guianensis* and *Siparuna gesnerioides* obtained with Aedes aegypti. Dashed lines represent the expected mortality based on the estimation for *A. aegypti* larvae. Significance levels (Z test, *p* < 0.05) are shown for each of concentration (i.e., LC_25_, LC_50_, and LC_80_). ns means the absence of statistically relevant differences. Mortality was recorded over a 24 h period.

Nymphs of *B. anurum* were adversely affected when exposed to *Siparuna* essential oils (Figure 5). *Siparuna gesnerioides* essential oil exhibited higher toxicity by killing over 83% of *B. anurum* nymphs at concentration as lower as 0.053 μg/mL, the estimated LC_25_ for *A. aegypti* larvae (Figure 5). Nymphs exposed to *S. gesnerioides* essential oil LC_50_ (0.070 μg/mL) exhibited a mortality rate of 86.7%, while 100% of nymphs were killed at this essential oil LC_80_ (0.109 μg/mL) (Figure 5). At low (LC_25_ = 0.063 μg/mL) and intermediary (LC_50_ = 0.078 μg/mL) concentrations, the *S. guianensis* essential oil toxicity to *B. anurum* nymphs did not differ to that recorded for mosquito larvae (Figure 5). However, when the exposure was at highest concentration LC_80_ (0.109 μg/mL), the mortality rate of 100.0% of *B. anurum* nymphs, which is significantly higher than the mortality estimated for the mosquito larvae (Figure 5).

## 3. Discussion

In this study, we demonstrated that while the essential oils of *Siparuna gesnerioides* and *S. guianensis* are sesquiterpene-rich, with germacrene D (in both essential oil types) and γ-elemene (only in *S. guianensis* essential oil) as major constituents. *S. gesnerioides* yielded a notably higher oil content (0.38%) compared to *S. guianensis* (0.21%). Both essential oils showed similar larvicidal activity against *A. aegypti*, though *S. guianensis* oil at LC_25_ exhibited greater lethality toward insecticide-resistant larvae. In contrast, *S. gesnerioides* essential oil was less selective to *B. anurum* nymphs, exhibiting higher lethality at lower concentrations (LC_25_), emphasizing the distinct selectivity of both essential oils. We further demonstrated, by means of an in silico analysis, that germacrene D and γ-elemene bind stably to *A. aegypti* AChE, suggesting a potential mode of action for these essential oils.

Plant-derived insecticides have re-emerged as viable alternatives in pest management, with essential oil of several *Siparuna* species showing toxic activity against agricultural and medically significant arthropods [16,17,25,26,27,29]. Our findings support the use *Siparuna* essential oils as biorational insecticides, particularly for controlling *A. aegypti* larvae, demonstrating that *S. gesnerioides* essential oil exhibited an LC_50_ values below 0.08 μg/mL. This larvicidal potency is within the range described for other *Siparuna* essential oils [17,28,38] and aligns with findings described elsewhere [39] noting that only 6% of essential oils tested (337 essential oil types from 225 plant species) as larvicides achieved LC_50_ values below 10 μg/mL. The essential oil yield (0.38%) for *S. gesnerioides* is within the range (i.e., between 0.10 and 0.61%) reported for other *Siparuna* essential oils [25,40,41,42].

Differences in essential oil composition between species, despite similar environmental conditions, may stem from genetic or physiological factors, as has been observed in other plants [16,43]. Our efforts represent the first phytochemical description of the essential oil of *S. gesnerioides*. The concentrations of the main constituents in our *S. guianensis* essential oils differ from those reported in previous studies [16,17,28,44], which also found variations in the amounts of the major compounds. Furthermore, while *S. guianensis* and *S. gesnerioides* essential oils share four compounds, including the major constituent germacrene D, some distinctions (e.g., the absence of γ-elemene in *S. guianensis* essential oil) may help to explain the recorded differences in mortality rates for *A. aegypti* and the non-target water bug *B. anurum.* These changes in the composition and concentration of the *S. guianensis* and *S. gesnerioides* essential oils may be due to plant physiological and genetic aspects since the two species were collected in the same area.

Although the main modes of action are not fully understood, comprehensive reviews have described that terpene-rich essential oils can disrupt the functions of ligand (e.g., octopamine, tyramine, and GABA)-activated receptors [45,46,47,48,49,50], non-selective ion (transient receptor potential—TRP) channels [27,51,52,53], and inhibiting the activities of acetylcholinesterase [54,55]. In this study, our molecular predictions indicated that both major constituents of *S. guianensis* and *S. gesnerioides* essential oils, germacrene D and γ-elemene, stably bind to *A. aegypti* AChE, suggesting that these molecules may disrupt the functions of these enzymes, adding insight into the mode of action of these *Siparuna* essential oils. Indeed, germacrene D, a common sesquiterpene in *Siparuna* as in other plant species, has shown to mediate plant–insect interactions and a broad insecticidal activity [56,57,58], disrupting the AChE and TRP channel functions in target and non-target organism [51,59]. Regarding γ-elemene, several studies have shown its insecticidal/repellent activities [60,61,62,63], resulting in its inclusion on patented formulations [64]. However, our work provides the first direct evidence of potential molecular targets for γ-elemene in insects. It is worth noting that our efforts do not exclude the potential involvement of other constituents presented in minor concentrations, as recent investigations have shown that insecticidal/repellent activities often depend not on the most abundant compound but on specific interactions among oil constituents [65,66].

Despite the beneficial potential of essential oils from *S. gesnerioides* and *S. guianensis* for controlling mosquito larvae, and contrasting with previous investigations that have shown the low toxicity of *Siparuna* essential oils to non-target organisms [25,26,27,29], our findings indicate these plant-based biorational tools can also be toxic to non-target aquatic arthropods such as the nymphs of *B. anurum*. Such findings reinforce the necessity for the careful assessment of the unintended impacts associated with the use of plant-based biorational pesticides used for controlling mosquito larvae [12,13]. In contrast to our findings, previous investigations on larvae [35] have reported no lethality in *B. anurum* when exposed to extracts from *Chiococca alba*, even at concentrations capable of killing 80% of *A. aegypti* larvae, indicating that those extracts exhibited higher selectivity towards the predatory insects when compared to the essential oils tested here.

## 4. Materials and Methods

### 4.1. Collection of Plant Material and Extraction of Essential Oil

Leaves of S. *guianensis* and S. *gesnerioides* were collected in the municipality of Norcasia (5°34′27″ N 74°53′20″ O; altitude 700 m), Caldas State, Colombia. Taxonomic identification was confirmed by experts at the herbarium (Universidad de Caldas, Manizales, Caldas State, Colombia), where samples were deposited with reference numbers JAO 957 (*S. guianensis*) and JASG 1522 (*S. guianensis*). For each species, we collected young and old leaves from different parts of the plants, ensuring the randomness of the sample. The material was placed in paper bags, identified, and transported to the Zoology Laboratory of the Universidad de Caldas (Manizales, Colombia). The leaves were dried in the shade at room temperature. After drying, the leaves were placed in plastic bags and stored until the extraction of the essential oil. The essential oils were obtained in the Kupay laboratory (Bogota–Cundinamarca, Colombia) by steam distillation. Briefly, the essential oils were extracted using a steam distillation unit (10 Kg) that resembles those used by commercial distillers. In the extractions, about 1600 g of dried leaves were used and the distillation time was four hours.

### 4.2. Chemical Composition of the Essential Oil

The identification and quantification of essential oil constituents were performed using gas chromatography with a flame ionization detector (GC-FID, QP2010SE, Shimadzu, Japan) and gas chromatography coupled to mass spectrometry (GC-MS, QP2010SE, Shimadzu, Japan) according to the methodology of [67]. For these characterizations, the following conditions were adopted: the carrier gas used was helium (He) for both detectors with flow rate and linear velocity of 2.80 mL min^−1^ and 50.80 cm s^−1^ (GC-FID) and 1.98 mL min^−1^ and 50.90 cm s^−1^ (GC-MS), respectively. The injector temperature was 220 °C at a split ratio of 1:30; fused silica capillary column (30 m × 0.25 mm); Rtx^®^-5MS stationary phase (0.25 μm film thickness). The oven temperature had the following schedule: initial temperature of 40 °C, which remained for 3 min, and then the temperature was gradually increased at a rate of 3 °C min^−1^ until it reached 180 °C, where it remained for 10 min, having a total analysis time of 59.67 min. The temperatures that were used in the flame ionization (FID) and MS detectors were 240 and 200 °C, respectively. The samples used were drawn from the vial in a volume of 1 μL of a 1% solution of EO in 95% hexane.

Analyses using gas chromatography coupled to mass spectrometry (GC-MS) were performed in an electron impact device with an energy of 70 eV; scan rate of 1000; scan interval of 0.50 fragments s^−1^; and detected fragments from 29 to 400 (m/z). Analyses using gas chromatography with a flame ionization detector (GC-FID) were performed with a flame formed by H_2_ and atmospheric air with a temperature of 300 °C. Flow rates of 40 mL min^−1^ and 400 mL min^−1^ were used for H_2_ and air, respectively. The identification of the components of the essential oils was performed by comparing the mass spectra obtained with those available in the spectrophotometer database (Wiley 7, NIST 05, and NIST 05s) and by the retention index (IR). For the IR calculation, a mixture of saturated C7-C40 alkanes (Supelco Inc., Bellefonte, PA, USA) submitted under the same chromatographic conditions as the essential oil was used, and the adjusted retention time of each compound was obtained using GC-FID. Then, the calculated values for each compound were compared with those in the literature [68,69,70]. The relative percentage of each compound in the EO was calculated by the ratio of the integral area of the peaks to the total area of all constituents in the sample with a relative area above 0.5%.

### 4.3. Aedes aegypti and Belostoma Anurum Rearing Conditions

We used *A. aegypti* larvae (fourth instar—L4) of the insecticide-susceptible (PPCampos) and insecticide-resistant (Oiapoque) strains that have been reared under controlled conditions in an insecticide-free environment over eight years. The larvae were kept in dechlorinated water, being fed daily with turtle food at a controlled temperature (temperature: 25 ± 2 °C; relative humidity: 60 ± 2%; photoperiod of 12 h of light) until the L4 stage, which is ideal for conducting larvicidal activity assays, following the methodology previously described in [7].

Nymphs of *B. anurum* were obtained from a laboratory strain that has been kept in an insecticide-free environment for over a decade. The initial strain consisted of individuals collected from fish farming facilities at the Federal University of Viçosa (UFV, Viçosa, MG, Brazil, 20°45′ S, 42°52′ W). Field-collected adult insects were maintained in a plastic pot (2L) in pairs (1 male and 1 female) with 1L of dechlorinated water under controlled conditions of a temperature of 25 ± 2 °C and a photoperiod of 12:12 L:D. Each aquarium had the presence of water hyacinth plants (*Eichhornia crassipes* (Mart.)), which were used as a resting and mating shelter. After mating, males with egg pads on their backs were kept in the plastic pot until hatching. After hatching, the first instar nymphs were individualized in glass containers (15 mL) with 10 mL of dechlorinated water to avoid cannibalism. The specimens were fed daily with *A. aegypti* larvae (L4) until the nymphs reached the second instar, which was used in bioassays.

### 4.4. Larvicidal Activity of Essential Oils Against Aedes Aegypti Larvae

We evaluated the larvicidal activity of *S. gesnerioides* essential oil in *A. aegypti* larvae (L4) of PPCampos (insecticide susceptibility pattern) strain, comparing its potential with the larvicidal activity recorded for the *S. guianensis* essential oil. For each essential oil type, we used at least five concentrations ranging from 0.033 μg/mL up to 0.196 μg/mL for *S. guianensis* essential oil, and from 0.037 μg/mL up to 0.131 μg/mL for *S. gesnerioides* essential oil. These essential oil concentrations were chosen based on a preliminary test to discover the smallest concentration capable of killing 100% of larvae and the highest concentration that did not kill any larvae tested. We always used dimethylsulfoxide (DMSO) at a concentration of 3.33 μg/mL to dilute the essential oils. Groups of 25 larvae were separated using a Pasteur pipette and subsequently distributed into glass vials (250 mL of volumetric capacity) containing 50 mL of the essential oil-containing solutions, which consisted in our experimental unit. Our control treatment consists of groups of 25 larvae subjected to solutions (50 mL) containing only DMSO (3.33 μg/mL). For each essential oil concentration and control treatment, we used four replicates, totalizing 100 larvae per treatment. Mortality was recorded after a 24 h exposure period. Larvae that did not show movement or response to stimulation with a Pasteur pipette were considered dead.

We further evaluated the potential of both *S. guianensis* and *S. gesnerioides* essential oils to kill larvae of insecticide-resistant (Oiapoque) strain. We subjected these insecticide-resistant larvae to the estimated LC_25_ (*S. guianensis*: 0.067 μg/mL; *S. gesnerioides*: 0.056 μg/mL) and LC_80_ (*S. guianensis*: 0.112 μg/mL; *S. gesnerioides*: 0.109 μg/mL) of the essential oils for the individuals of the PPCampos strain. The exposure to the LC_25_ evaluates the lethality at lower concentrations, while the LC_80_ represents the minimum insecticide concentration required by regulatory agencies for toxicological tests aimed at characterizing insecticide efficacy. The exposure procedures were identical to those described above. For each treatment, we used 100 fourth instar larvae (i.e., four groups of 25 larvae as replicates). Mortality was assessed at 24 h after the start of the experiment. All toxicity bioassays were conducted at controlled temperature (25 ± 2 °C), humidity (60 ± 2%), and photoperiod (12 h light phase).

### 4.5. Molecular Interactions Between the Essential Oil Major Constituents (Germacrene D and Γ-Elemene) and Acetylcholinesterases (AChEs) of Aedes Aegypti

We used in silico approaches to predict potential molecular interactions of the essential oil major constituents (germacrene D and γ-elemene) and the acetylcholinesterases (AChEs) of *A. aegypti*. Amino acid sequences of AChE of *A. aegypti* were retrieved from the National Center for Biotechnology Information (NCBI) database. The 3D structures of proteins were constructed by modeling approach using Swiss Model Workspace “https://swissmodel.expasy.org (accessed on 24 January 2025)” with the template of X-ray crystal structure of PDB code 6XYY for the *A. aegypti* AChE. The clashes in crystallographic structures and amino acid positioning in the active site were checked using Swiss model [71]. The validation of the generated models was performed by analyzing the Ramachandran plot [72,73], and the QMEAN factor was also analyzed [74].

To predict docking calculations, germacrene D and γ-elemene ligands were downloaded from PubChem [75] at National Institutes of Health (NIH) and saved in sdf format. Additionally, the ligands and AChE protein were prepared using Autodock Tools [76] adding charges, polar hydrogens, selecting grid to the protein and saving all molecules in pdbqt format [77]. Docking positions were generated for the germacrene D and γ-elemene interacting with AChE of *A. aegypti*, returning affinity energy values (kcal [4.184 kJ]/mol) using the AutoDock Vina [78]. The best position for each ligand–protein complex was used for generating 2D interaction maps with Discovery Studio [79].

### 4.6. Essential Oil Toxicity on Nymphs of Belostoma anurum

We exposed the 2nd instar nymphs of *B. anurum* to essential oils concentrations estimated to the PPCampos larvae. We used the *S. guianensis* concentrations of 0.063 μg/mL; 0.078 μg/mL; 0.112 μg/mL, which corresponds to the LC_25_, LC_50_, and LC_80_ values. Similar concentrations (LC_25_ = 0.053 μg/mL; LC_50_ = 0.070 μg/mL; LC_80_ = 0.109 μg/mL) were used for the *S. gesnerioides* essential oil. In order to avoid cannibalism, nymphs were individually exposed to the essential oils, where each of them was placed into a glass vial (20 mL) having 15 mL of the essential oil-containing solutions. Control individuals were exposed to DMSO (3.33 μg/mL)-containing solutions. For each treatment, we used 30 nymphs (i.e., three groups of 10 individualized nymphs as replicates). Mortality was assessed at 24 h after the start of the experiment. All toxicity bioassays were carried out under controlled conditions of temperature (25 ± 2 °C), humidity (60 ± 2%), and photoperiod (12 h light phase).

### 4.7. Statistical Analysis

The concentration-mortality results obtained in the toxicological bioassays with *A. aegypti* larvae were subjected to a Probit analysis using the PROBIT procedure in the SAS 9.2 statistical software platform (SAS Institute, Cary, NC, USA, 2008). The mortality of the insecticide-resistant larvae and *B. anurum* nymphs were subjected to analysis of variance (ANOVA) and compared by Tukey’s HSD test (*p* < 0.05) using SigmaPlot 14.0 (Systat Software, San Jose, CA, USA).

## 5. Conclusions

Our findings demonstrate that *S. gesnerioides* and *S. guianensis* possess comparable larvicidal activities, highlighting the potential of *Siparuna* essential oils as effective, environmentally friendly alternatives for integrated pest management. Notably, the increased toxicity of *S. guianensis* against *A. aegypti* larvae of a strain shown to exhibit resistance to insecticide larvae reinforces its promise as a strategy to combat resistance in mosquito populations. Furthermore, the molecular insights into the interaction of γ-elemene with AChE enhance our understanding of the mechanisms underlying the insecticidal effects of these essential oils, contributing valuable information for the development of natural pest control agents. However, the recorded lower selectivity of both *Siparuna* essential oils against *B. anurum* nymphs emphasizes the necessity of conducting thorough risk assessments for plant-based biorational insecticides. This challenge calls for the development of optimized formulations that can maximize the efficacy of *Siparuna* essential oils while minimizing their toxicity to non-target organisms.

## Figures and Tables

**Table 1:** Chemical composition, concentrations (%), and terpene classification for the essential oil of *Siparuna guianensis* and *Siparuna gesnerioides*.

N°	Compound	Terpenic Classification ^a^	*Siparuna guianensis*		*Siparuna gesnerioides*	
			t_retention_	A_rel_ (%)	t_retention_	A_rel_ (%)
1	α-copaene	HS	28.632	4.7	28.636	6.7
2	β-bourbonene	HS	29.002	5.5	-	-
3	β-caryophyllene	HS	30.484	4.6	30.479	6.6
4	germacrene D	HS	33.212	31.9	33.263	43.8
5	γ-elemene	HS	33.924	45.8	-	-
6	Δ-cadinene	HS	35.046	7.5	35.068	11.4
7	β-pinene	HM	-	-	10.318	3
8	α-bergamotene	HS	-	-	31.306	10.7
9	α-humulene	HS	-	-	31.927	3.7
10	aromadendrene	HS	-	-	33.383	6.1
11	α-amorphene	HS	-	-	34.586	2.8
	Total Identified		100.0		94.8	

^a^ Terpenic classification: hydrocarbon monoterpene (HM) and hydrocarbon sesquiterpene (HS).

**Table 2:** Toxicity of essential oils of *Siparuna guianensis* and *Siparuna gesnerioides* to fourth instar larvae (L4) of *Aedes aegypti* over a 24 h exposure period. The lethal concentration (LC) values are expressed in μg/mL.

Plant Species	Slope ± SE	LC_25_ (95% CI)	LC_50_ (95% CI)	LC_80_ (95% CI)	Toxicity Ratio (TR_50_) *
*Siparuna guianensis*	5.42 ± 0.56	0.063 (0.060–0.067)	0.078 (0.074–0.083)	0.101 (0.094–0.112)	-
*Siparuna gesnerioides*	5.55 ± 0.43	0.053 (0.049–0.056)	0.070 (0.066–0.074)	0.099 (0.091–0.109)	1.09 (0.95–1.21)

* TR_50_ was calculated by dividing LC_50_ obtained for *S. guianensis* per LC_50_ obtained for *S. gesnerioides*. The toxicity ratio is considered significant if it does not include the value of 1.0 [37].

## Data Availability

The data presented are available on request from the corresponding authors.
